# Marked stem cell factor expression in the airways of lung transplant recipients

**DOI:** 10.1186/1465-9921-7-90

**Published:** 2006-06-16

**Authors:** Carla A Da Silva, Mélanie Adda, Marc Stern, Frédéric de Blay, Nelly Frossard, Dominique Israel-Biet

**Affiliations:** 1 1EA 3771 'Inflammation and environment in asthma'. Faculté de Pharmacie, BP 60024, 67401 Illkirch Cedex, France; 2 UPRES EA 220. Université Paris V. UFR Biomédicale des Saints-Pères, 45 rue des Saints-Pères, 75006 Paris, France; 3 Service de Pneumologie. CMC Foch, 40 rue Worth, 92151 Suresnes Cedex, France; 4 Service de Pneumologie. Hôpital Européen Georges Pompidou, Faculté de Médecine Paris V, 20 rue Leblanc, 75015 Paris, France

## Abstract

**Background:**

Airways repair is critical to lung function following transplantation. We hypothesised that the stem cell factor (SCF) could play a role in this setting.

**Methods:**

We studied 9 lung transplant recipients (LTx recipients) during their first year postgraft, and evaluated SCF mRNA expression in bronchial biopsy specimens using on-line fluorescent PCR and SCF protein levels in bronchoalveolar lavage (BAL) and serum using ELISA. The expression of SCF receptor Kit was assessed using immunostaining of paraffin-embedded bronchial sections.

**Results:**

SCF mRNA was highly expressed during the early postgraft period [Month (M)1-M3] (300% increase vs controls: 356 vs 1.2 pg SCF/μg GAPDH cDNA, *p *< 0.001) and decreased thereafter (M4-M12: 187 pg/μg), although remaining at all times 10–100 times higher than in controls. While SCF protein levels in BAL were similar in LTx recipients and in controls, the SCF serum levels were at all times higher in LTx recipients than in controls (*p *< 0.05), with no relationship between these levels and the acute complications of the graft. Finally, Kit was strongly expressed by the mast cells as well as by the bronchial epithelium of LTx recipients.

**Conclusion:**

SCF and Kit are expressed in bronchial biopsies from lung transplant recipients irrespective of the clinical status of the graft. A role for these factors in tissue repair following lung transplantation is hypothesised.

## Background

Injury processes of various types can damage a grafted organ. Some are due to the surgical procedure itself-the section of vessels and nerves and, for lung transplants, conducting airways. Others are inflammatory in nature, due to reperfusion of the graft or early allogeneic reactions. The lack of efficient tissue repair mechanisms would severely impair graft functioning. The restoration of transplanted airways involves a variety of cell types: local progenitors, found mainly among basal and Clara cells [1-3], and host stem cells from the bone marrow, a rich reservoir of progenitors for different cell types including mast cells [4-6]. Experimental studies in murine models provide evidence that bone marrow stem cells can differentiate into type I and type II pneumocytes and bronchial epithelial cells [5,7]. The ability of such cells to engraft into the human lung is still controversial although studies after hematopoietic stem cell transplantation in humans show evidence of donor-derived cells (chimerism) in the lung [8,9]. In this context, SCF is a key factor in mobilisation of stem cells from bone marrow where it facilitates egress from the marrow to the circulation [10]. On the other hand, there is some evidence for the involvement of local progenitor cells in repair processes following tissue injury in murine models [11-13] as well as in human situations [14,15]. The SCF/Kit pathway has also been shown to play a key role in these latter.

To our knowledge, SCF production has never been evaluated in the lung in settings other than asthma, where it is markedly upregulated [16]. More specifically, it has never been evaluated in the context of solid organ transplantation. We hypothesised that it might be produced soon after transplantation to participate in airway tissue repair. We therefore evaluated SCF mRNA expression in bronchial biopsy specimens and SCF protein expression in bronchoalveolar lavage (BAL) and serum during a one-year follow-up of human LTx recipients. We also assessed SCF receptor Kit expression in bronchial epithelium using immunodetection. Finally, because SCF is a well-known mast cell growth factor (for review see [17,18]), we also quantified the number of mast cells infiltrating the airways.

## Methods

### Study population

Nine (3 single and 6 double) lung transplant recipients (3 males, 6 females), aged 41 (median; range: 22–60 years), were included into this study. Their initial diagnoses were cystic fibrosis (n = 3), emphysema (n = 2), diffuse bronchiectasis (n = 2), pulmonary artery hypertension associated with systemic sclerosis (n = 1), and lymphangioleiomyomatosis (n = 1). All patients were initially placed on an immunosuppressive regimen including cyclosporin A, azathioprine and prednisone, until the third episode of acute lung rejection (ALR) after which cyclosporin A was switched to tacrolimus and azathioprine to mycophenolate mofetil. In addition to clinical and functional evaluation, fiberoptic bronchoscopy with bronchoalveolar lavage (BAL), transbronchial biopsies and proximal bronchial biopsies were routinely performed in each patient either to detect asymptomatic complications at day 30 after surgery (then every month during the first 6 months and every 6 months thereafter) or as part of the routine procedure for the diagnosis of complications suspected on clinical and/or functional grounds. One patient died 8 months after the transplant of infectious (Aspergillus) complications. None of the patient was diagnosed with bronchiolitis obliterans syndrome (BOS) during the study period but 4 of them subsequently developed this complication between 15 and 33 months post-transplantation, leading to death in two.

Control subjects (4 males and 5 females, aged 25 (median; range: 21–38 years) were all healthy volunteers. The study was approved by the local Ethics committee, and all patients and control subjects provided written informed consent.

### Tissue specimens

Bronchial biopsy specimens were fixed in 10% buffered formalin and paraffin-embedded. They were cut into four- μm sections for morphological and immunohistochemical evaluation. An additional biopsy sample was snap-frozen in liquid nitrogen for subsequent RNA extraction. Four to six serial samples per patient, recovered during the first year post-transplantation, were evaluated.

### RNA extraction and reverse transcription

Total RNA was extracted from biopsy specimens with TriReagent^® ^(Molecular Research Center Inc., Cincinnati, OH, USA). Isolated RNA was diluted in RNase-free water and quantified by absorbance measurement at 260 nm. Two μg of total RNA were incubated with 0.5 μg of random primers for 5 min at 70°C, and allowed to cool down at room temperature. RNA was subsequently reverse-transcribed in 1× reverse transcription (RT) buffer (75 mM KCl, 3 mM MgCl2, 10 mM dithiothreitol, and 50 mM Tris-HCl, pH8.3), containing 1 unit/μl RNasin ribonuclease inhibitor, 1 mM of each dNTP, and 10 units/μl RNase H(-)-Moloney leukemia virus reverse transcriptase (all reagents from Promega, Madison, WI, USA). The reaction was conducted for 1 h at 37°C, and then the reverse transcriptase was heat-inactivated at 99°C for 5 min.

### Quantification of SCF cDNA

Reverse transcribed cDNAs were amplified by on-line fluorescent PCR (LightCycler™-SYBR GreenI, Roche Diagnostics), with primers leading to single 149-bp and 240-bp PCR products for quantification of SCF and GAPDH (used as a housekeeping gene) cDNAs, respectively. PCR reactions were performed in 1× PCR reaction buffer [2 μl of the reaction mix, containing FastStart Taq DNA polymerase, dNTP mix, SYBR Green I, 3 mM MgCl_2 _(Roche Diagnostics)], and 10 pmol of each primer:

SCF amplification: sense primer 5'-TGGATAAGCGAGATGGTAGT-3'antisense primer 5'-TTTTCTTTCACGCACTCCAC-3', GAPDH amplification:sense primer 5'-GGTGAAGGTCGGAGTCAACGGA-3' antisense primer5'-GAGGGATCTCGCTCCTGGAAGA-3', in a 20 μl final volume for 35 cycles. Each cycle consisted of 15 sec denaturation at 95°C, 10 sec annealing (at 53°C for SCF cDNA amplification and 60°C for GAPDH) and 10 sec extension at 72°C. Amplified SCF and GAPDH cDNAs and standard SCF and GAPDH cDNAs were analysed on-line by fluorescence (LightCycler™, Roche Diagnostics).

The standard SCF and GAPDH cDNA came from pulmonary fibroblast total cDNA. After amplification by PCR, with the primers described above, the 149 bp- and 240 bp-PCR products were electrophoresed on a 2% agarose gel, stained with ethidium bromide, purified on QIAEX II (QIAEX II gel extraction kit, QIAGEN, Courtaboeuf, France), and quantified by fluorescence (PicoGreen^®^, Molecular Probes Inc.) according to a standard curve obtained with a double-stranded phage λ DNA (0.005–1 μg/ml). The purified SCF cDNA and GAPDH cDNA were used to establish a standard curve from 1 to 300 fg/ml.

Quantification of SCF cDNA (pg) was normalised to the GAPDH cDNA measurement. Results were expressed as pg SCF cDNA/μg GAPDH cDNA.

### Soluble SCF protein measurement in BAL and serum

A sensitive ELISA procedure quantified immunoreactive SCF released into patients' BAL fluid and serum. It used a capture anti-human SCF monoclonal antibody (R&D Systems Europe, Abingdon, UK, clone 13302) and an anti-human SCF biotinylated polyclonal antibody (R&D Systems Europe), revealed by extravidin-horseradish peroxidase and a 3,3',5,5'-tetramethylbenzidine liquid substrate system (Sigma Chemicals, St Louis, MO, USA). Standard curves were generated with recombinant human SCF (R&D Systems Europe) diluted in fœtal calf serum. They were linear from 16 to 500 pg/ml. Eight normal blood donors provided serum for control purposes. Soluble SCF concentrations were expressed as pg/ml of BAL fluid or serum.

### Kit immunodetection

Four- μm sections of paraffin-embedded biopsies were cut and used for immunohistochemistry. Briefly, tissue sections previously deparaffinised in toluene and rehydrated through graded concentrations of ethanol, were incubated for 60 min at room temperature with a primary polyclonal rabbit anti-human CD117 (Kit) (1/100) (Santa Cruz Biotechnology, Inc; CA, USA). After two washings with Tris buffer at pH 7.6, staining was revealed according to the avidin-biotin method using a LSAB AP kit (Dako Corp; Carpinteria, CA, USA) and FastRed as a substrate. Tissue sections were finally counterstained with hematoxylin and mounted with Ultramount medium (Dako Corp; CA, USA). To ensure the anti-Kit binding specificity, two types of negative controls were used: 1) by omitting the primary antibody and 2) by preincubating the primary antibody with a specific blocking peptide (sc-168 P, Santa Cruz Biotechnology, Inc; CA, USA).

### Mast cell counts

The immunodetection of mast cells was similar to that described above with the primary antibody being a monoclonal mouse anti-human tryptase (0.25 μg/ml, Dako Corp; CA, USA). Results are expressed as the number of tryptase-positive cells per mm^2 ^of bronchial structure.

### Statistical analysis

All results are expressed as median values (SCF mRNA, SCF protein levels, mast cell numbers) collected during two different periods of time: M1-M3 (months 1 to 3 postgraft, during which the immunosuppressive regimens are usually the most intensive) and M4-M12 (months 4 to 12 post-graft, during which corticosteroids doses are usually tapered in case of an uneventful evolution of the graft). Comparisons were made using the non-parametric Mann Whitney U-test for unpaired series. A *p *value <0.05 was considered significant.

## Results

### SCF mRNA

**Figure 1:**
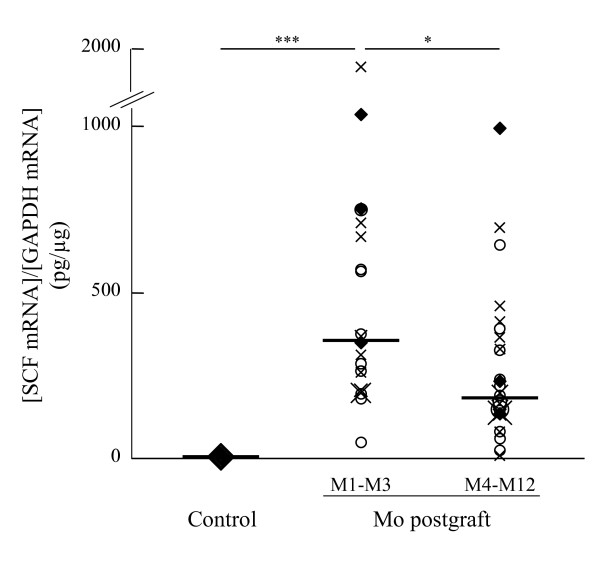
SCF mRNA expression in lung transplant recipients. SCF mRNA was quantified after total RNA reverse transcription by on-line fluorescent PCR in biopsy specimen from control subjects (Control) and lung tranplant patients at Months (Mo) postgraft 1 to 3 (M1-M3), and 4 to 12 (M4-M12). Results were normalised to GAPDH mRNA expression, and expressed in pg/μg as the ratio [SCF mRNA]/[GAPDH mRNA]. Symbols denote individual episodes (◆ : stable condition, ○ : acute lung rejection, × : infection). Median values are also shown (bold line) (NS; non-significative, *; *p *< 0.05, **; *p *< 0.01, ***; *p *< 0.001).

High levels of SCF mRNA expression were observed in the airways of every lung transplant recipient. This expression was particularly marked during the early post-transplant period (M1-M3), when it was 300% of the level in controls (356 pg SCF/μg GAPDH (range: 59–1826) in patients and 1.2 pg SCF/μg GAPDH mRNA (range: 0.4–5.8) in controls, *p *< 0.001) (Figure 1). SCF expression thereafter decreased in lung transplant recipients (M4-M12: 187 pg/μg GAPDH mRNA (range: 10–987), *p *< 0.05 *vs *M1-M3) but remained 10–100 times higher in patients than controls throughout this period (Figure 1). We found no statistical relation between clinical status and SCF mRNA expression, which remained at comparable levels when the patient's condition was stable and during acute complications.

### SCF protein

**Figure 2:**
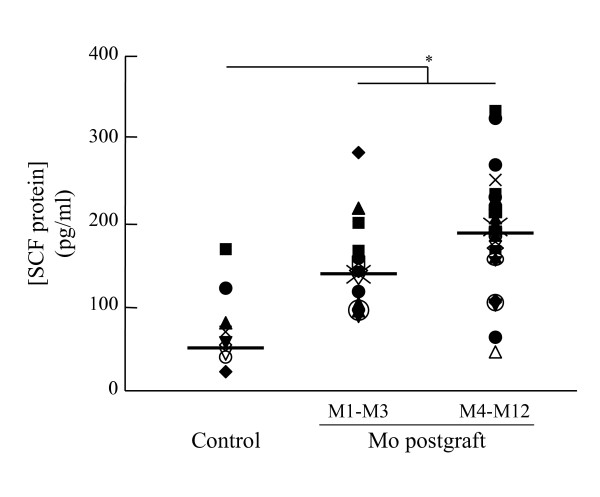
SCF protein levels in serum. SCF protein levels were assessed by ELISA in serum from control subjects (Control) and lung transplant recipients at Months (Mo) postgraft 1 to 3 (M1-M3), and 4 to 12 (M4-M12). Results are expressed as pg/ml, and represented as individual (symbols) and median values (bold line) (NS; non-significative, *; *p *< 0.05, **; *p *< 0.01, ***; *p *< 0.001).

It was detected in every BAL sample taken from LTx recipients. Its concentration remained comparable to controls throughout the study period (70 pg/ml (range: 34–182) during M1-M3, 67 pg/ml (range: 35–144) during M4-M12 and 58 pg/ml (range: 1–90) in controls, NS for all comparisons). In contrast, serum SCF levels remained at all times higher in LTx recipients than in controls (142 pg/ml (range: 104–265) during M1-M3, 193 pg/ml (range: 67–338) during M4-M12 and 53 pg/ml (range: 28–174) in controls, *p *< 0.05 controls vs both post-transplant periods) (Figure 2).

### Kit expression

**Figure 3:**
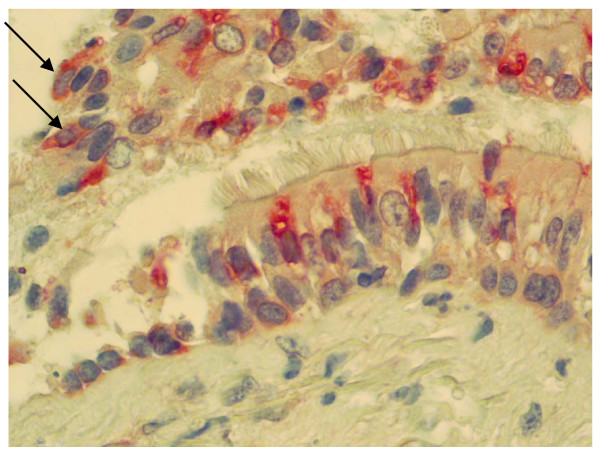
Photomicrograph of Kit expression in LTx recipients biopsy sections. Kit was immunodetected using a polyclonal rabbit anti-human CD117 (Kit) in bronchial biopsy specimen from LTx recipients in mast cells (arrow head) and epithelial cells (magnitude ×40).

SCF receptor Kit could be evaluated in 5 of 9 LTx recipients. Figure 3 shows a marked staining of the mast cells as well as of the bronchial epithelium with a clear immunolocalisation on some basal and some ciliated epithelial cells (Figure 3).

### Tryptase-positive cells

**Figure 4:**
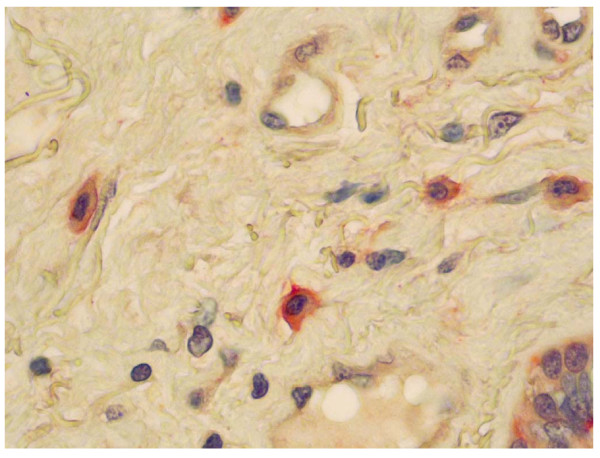
Tryptase-immunoreactive mast cells in the bronchial biopsy sections from Ltx patients. Mast cell immunolabelling was performed using a mouse monoclonal antibody raised against human tryptase and revealed by FastRed^® ^in lung biopsy specimen from lung transplant recipients (magnitude ×40).

Non degranulated tryptase-positive cells were observed in almost all biopsy specimens from LTx recipients. They were mainly located in the bronchial submucosa but also infiltrated the bronchial epithelium (Figure 4). In the early post-transplant period, mast cell counts were not different in patients and controls (36 (range: 0–215) *vs *30 (range: 7–71)/mm^2 ^in the M1-M3 period and in controls, respectively; NS). They subsequently increased significantly in LTx recipients: 71 (range 2–224)/mm^2 ^during M4-M12 (*p *< 0.05 vs M1-M3 and vs controls). This increase appeared independent of patients' clinical condition (stable condition or acute complications).

## Discussion

Our study shows that SCF was highly expressed in 9 of 9 lung transplant recipients irrespective of their clinical status (infection, rejection). The level of this expression is remarkable, being 300-times greater than in controls in the early post-graft period and albeit decreasing thereafter, remaining at least 10-fold over the control values until the end of the first year post-transplant. To our knowledge, such high levels of SCF transcripts have never been reported so far in human airways. They are striking given the high corticosteroid doses administered to the patients during the early post-transplant period, knowing the usual inhibition of SCF expression by this drug as reported in asthma patients treated with glucocorticoids [16] as well as in pulmonary fibroblasts *in vitro *[19]. The only inflammatory airway condition in which SCF expression been reported before is asthma [16,20], albeit with levels largely lower than those reported in this study. None of the underlying diseases which indicated pulmonary transplantation in our patients has ever been reported to be associated with SCF airway expression.

With respect to SCF protein, although it was detected in the BAL fluid of every LTx recipients, it remained at levels comparable with those of controls. In contrast, SCF serum levels were significantly higher in LTx recipients than in controls. One possible explanation for the apparent discrepancy between SCF BAL and serum levels is that inflammatory alveolar cells, the main cell compartment sampled by BAL, are not its main source in the transplanted lung. Although our study does not allow us to draw conclusions about the nature of producing cells, we can hypothesise that bronchial cells are also at least partly involved in this production since all resident lung cells have this ability i.e. normal epithelial cells, smooth muscle cells and fibroblasts [20-22].

The role of SCF in this context can only be speculative. In our small cohort of patients, we found no relationship between mRNA levels and the current clinical situation, particularly acute complications of the graft. It was highly and early expressed even in subjects in stable condition. One hypothesis is that the SCF produced locally might play a role in inflammatory/repair processes following transplantation. Along with this hypothesis, it is of interest to note the expression of the SCF receptor Kit, which we have shown in the bronchial epithelium of LTx recipients. The SCF/Kit pathway, functioning in an autocrine and/or paracrine manner, has clearly been implicated in repair processes taking place after different types of tissue injury in animal models [11-13] as well as in humans [14,15]. Alternatively, SCF having the ability to mobilise stem cells of hematopoietic origin [10,23], it could participate in their recruitment to the lung, in keeping with a recent experimental model of lung injury showing a partial pulmonary reconstitution by originally hematopoietic cells [6]. Indeed, some allografts exhibit a low but consistent chimerism [24-26]. This phenomenon has been recently confirmed in human lung allografts [27,28]. SCF might be one of the factors involved in the mobilisation and homing of hematopoietic lung progenitors to the injured/repairing zone. On the other hand, SCF is the main chemotactic, activation and growth factor for mast cells [17,18]. Of note is the fact that we have shown a certain degree of mast cell infiltration of transplanted airways particularly during the second post-transplant period (M4-M12). This delay might be partly explained by the high doses of corticosteroids included in the early postgraft immunosuppressive regimens, to which mast cells are known to be highly sensitive. In any case, mast cells can exert strong beneficial effects in inflamed organs through the release of a wide range of mediators involved in different aspects of wound healing [29-31]. Conversely, they have also been implicated in various fibrotic processes [32-35] as well as in chronically rejected lungs [36,37].

## Conclusion

Whether mast cells, which infiltrate the transplanted lung, are innocent bystanders attracted by potent chemotactic factors or are actively recruited for specific purposes is presently unknown. Only the continued follow-up of our patients and the repeated evaluation of these factors over time will tell us about the potential relationship between SCF expression, mast cell presence in the airways and lung transplant outcome.

## Abbreviations

ALR: Acute Lung Rejection

BAL: BronchoAlveolar Lavage

BOS: Bronchiolitis Obliterans Syndrome

LTx: Lung Transplant

SCF: Stem Cell Factor

## Authors' contributions

**CADS **performed SCF mRNA and protein measurements. Contributed to the writing of the paper. **MA **performed most of the mast cell immunolabeling and counting. Contributed to the writing of the paper. **MS **Head of the department of Pulmonary Medicine of the CMC Foch, where lung transplantation is performed. In charge of the entire clinical management of the patients, including the performance of fiberoptic bronchoscopies and bronchial biopsies. **FdB **Performed the bronchial biopsies. **NF **Head of the research team who performed mRNA and ELISA studies. Co-directed the study. Co-wrote the paper. **DIB **Head of the research team who performed immunohistochemistry. In charge of the immunological monitoring of lung transplant recipients referred above. Co-directed the study. Co-wrote the paper.
